# Water and nitrogen management effects on semiarid sorghum production and soil trace gas flux under future climate

**DOI:** 10.1371/journal.pone.0195782

**Published:** 2018-04-19

**Authors:** Benjamin D. Duval, Rajan Ghimire, Melannie D. Hartman, Mark A. Marsalis

**Affiliations:** 1 Department of Biology, New Mexico Institute of Mining and Technology, Socorro, NM, United States of America; 2 New Mexico State University, Agricultural Science Center, Clovis, New Mexico, United States of America; 3 Natural Resource Ecology Laboratory, Colorado State University, Fort Collins, Colorado, United States of America; 4 New Mexico State University, Agricultural Science Center, Los Lunas, New Mexico, United States of America; USDA Agricultural Research Service, UNITED STATES

## Abstract

External inputs to agricultural systems can overcome latent soil and climate constraints on production, while contributing to greenhouse gas emissions from fertilizer and water management inefficiencies. Proper crop selection for a given region can lessen the need for irrigation and timing of N fertilizer application with crop N demand can potentially reduce N_2_O emissions and increase N use efficiency while reducing residual soil N and N leaching. However, increased variability in precipitation is an expectation of climate change and makes predicting biomass and gas flux responses to management more challenging. We used the DayCent model to test hypotheses about input intensity controls on sorghum (*Sorghum bicolor* (L.) Moench) productivity and greenhouse gas emissions in the southwestern United States under future climate. Sorghum had been previously parameterized for DayCent, but an inverse-modeling via parameter estimation method significantly improved model validation to field data. Aboveground production and N_2_O flux were more responsive to N additions than irrigation, but simulations with future climate produced lower values for sorghum than current climate. We found positive interactions between irrigation at increased N application for N_2_O and CO_2_ fluxes. Extremes in sorghum production under future climate were a function of biomass accumulation trajectories related to daily soil water and mineral N. Root C inputs correlated with soil organic C pools, but overall soil C declined at the decadal scale under current weather while modest gains were simulated under future weather. Scaling biomass and N_2_O fluxes by unit N and water input revealed that sorghum can be productive without irrigation, and the effect of irrigating crops is difficult to forecast when precipitation is variable within the growing season. These simulation results demonstrate the importance of understanding sorghum production and greenhouse gas emissions at daily scales when assessing annual and decadal-scale management decisions’ effects on aspects of arid and semiarid agroecosystem biogeochemistry.

## Introduction

The enterprise of environmentally sustainable agriculture should address how agro-ecosystems respond to climate change as well as their contribution to climate change [1–4]. Understanding crop response to stress is also critical for making management decisions where increased stressors are likely and to develop strategies for insulating production from climate change. Cultivated areas of the southwestern United States experience high ambient temperatures, large diurnal temperature fluctuations, low, yet highly seasonal variable precipitation, high rates of evapotranspiration and high UV radiation inputs. These conditions are a significant challenge for agriculture that are likely to intensify under ongoing anthropogenic climate change [5]. The southwestern US may therefore be thought of as a testing ground for agriculture under climate extremes.

Growing regionally appropriate crops should be a first step in increasing agricultural sustainability [6, 7]. Corn (*Zea maize* L.) is the most cultivated crop by area in the contiguous United States [8] and constitutes the largest agricultural export from the US [9]. Corn’s outsized role in US agriculture is due to its use as animal feed, ethanol feedstock and inexpensive sugar. The widespread cultivation of corn has environmental costs that deserve consideration in making regionally appropriate agricultural decisions. Corn is highly fertilized, and tall tower measurements coupled with statistical inversion methods calculated direct N_2_O emissions from the Midwestern US Corn Belt to be 198 ± 80 Gg N in 2011 [10]. While there are multiple “omics” approaches to improving corn N efficiency [11], yield increases in corn have been followed by increases in drought sensitivity perhaps as a function of changes in agronomic practices over the past 30 years [12]. Numerous studies on biofuel systems use corn as a baseline to gauge performance of alternative crops. Studies evaluating switchgrass (*Panicum virgatum* L.), miscanthus (*Miscanthus x giganteus*) and sugar canes (*Saccharum spp*.) show trends toward lower greenhouse gas emissions and NO_3_^—^N loss, and higher values of soil quality when corn is replaced on a landscape [13–15]. Thus, the growing body of evidence that corn is not environmentally sustainable in the Corn Belt should caution against its cultivation in more stressful environments.

Sorghum is a C4 grass that can substitute for corn in many of its common applications in the US, such as animal feed [16], sugar [17] and grain for human consumption [18]. Indeed, sorghum is a staple crop in many African and south Asian countries, and likely evolved under many of the stress conditions associated with southwestern USA climate noted above [19]. Field trials with varieties grown for grain and forage production show that sorghum can thrive in semiarid environments, produce biomass on the order of Midwestern corn (5–25 Mg ∙ ha^-1^ dry mass in New Mexico); [20, 21], exhibits high nitrogen use efficiency [22], and can tolerate salinity [23].

Sorghum is relatively drought tolerant compared to other crops, however, ~ 30% of US sorghum hectares were irrigated in 2015 [8], and reports from New Mexico show significant yield increase in sorghum under irrigation [24]. Irrigated systems present a challenge for N management, as increased soil water creates favorable conditions for N loss through denitrification and leaching [25], necessitating greater N inputs to offset these losses. Comparing irrigated crops with dryland or rainfed counterparts often reveals a non-linear relationship between yield and plant water use [26], but N_2_O fluxes from sorghum show a positive relationship with irrigation [27] and high early season N_2_O fluxes following fertilization [28].

Split application of N fertilizer, or delaying applications to synchronize with crop demand, is a logical way to reduce N losses. In principal, this strategy minimizes N available for microbial transformation and losses to the atmosphere or through soil water flows [29, 30]. Split fertilization has been effective in producing higher yields with less N inputs to corn (Lewboski, personal communication), and is reported to lower yield-scaled N_2_O emissions [31]. A series of 28 experiments report no yield loss in corn with delaying N fertilization as late as the 11-leaf growth stage [32]. Experiments delaying or splitting N application to sorghum generally report no effects on yield [22, 33–35], but we are unaware of any studies that have also measured or modeled N loss from altering N application timing in sorghum.

**Fig 1:**
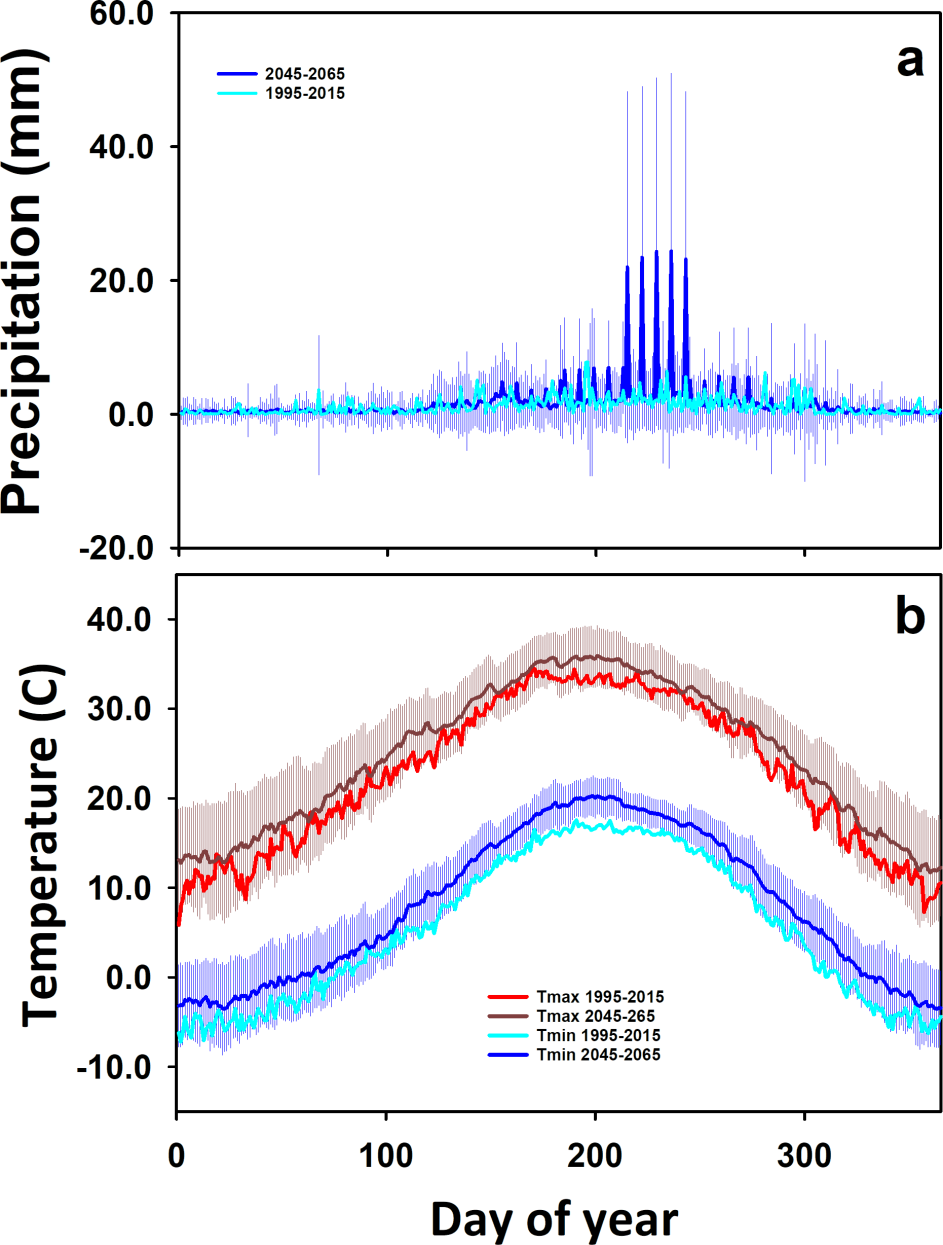
Current and future climate for eastern New Mexico. Climate inputs for DayCent simulation experiments of sorghum production. Data represent 20 year daily means from measured (1995–2015) and future downscaled (2045–2065) a) daily minimum and maximum temperature and b) precipitation. Error bars around future means are ± 1 standard deviation calculated from the range in daily values from the 5 models used in generating future weather.

Future weather projections for the southwest USA are for generally warmer, drier conditions [5]. The mean annual temperature in areas of New Mexico under both irrigated and dryland sorghum cultivation are forecast to increase by 2–4°C, with shifts in precipitation that do not necessarily reduce mean precipitation, but result in higher seasonal variability (Fig 1). Warmer temperatures perhaps reduce yield due to increased water vapor deficit [12] and shifts in precipitation could induce late season mortality or stunt grain development [36]. Precipitation shifts will also pose a challenge for predicting N loss and provide further motivation for efficient N management.

To test the hypothesis that selection of a drought adapted crop managed for N conservation can be environmentally sustainable under future climate, we define sustainability as biomass or emissions produced per unit of farm input. This approach is conceptually like life cycle analysis, because the cost of the product is considered in terms of inputs. We present the idea of input scaling in contrast to yield scaling emissions, because the latter can obfuscate the results of managing a system for emissions reduction. If a practice increases yield or biomass, and total emissions are flat or even slightly higher than an alternative practice, yield-scaled emissions give the appearance of emissions reduction. Scaling by input shows the quantity of product (grain, biomass) or emissions (N_2_O) per unit input. These are formally defined below as input-scaled biomass (ISB) and input-scaled emissions (ISE). As most sorghum production in the western US is for animal forage, we only consider biomass here and not grain yield.

We ran simulations with the biogeochemical model DayCent [37] to evaluate management intensity and future climate on a semiarid agro-ecosystem. We reported simulation results for sorghum production, trace gas flux and soil C dynamics in response to varying irrigation, N application rate and N application timing under current and future climate. We specifically tested the hypothesis that future production would be dependent on increased management intensity, which would be shown by a greater effect of management on biomass in the future compared to current climatic conditions. However, we also predicted that high variability in future precipitation will be a larger factor in estimates of N loss and soil C than specific management scenarios. We tested the hypothesis that irrigation reduces variation in biomass C production and gas emissions by stabilizing soil water. We further calculated management intensity as production and trace gas emissions per unit input (N or water addition) to define sustainability in terms of limiting resources.

## Materials and methods

### Sorghum field trial data

New Mexico State University administers a network of Agricultural Science Centers (ASC) throughout the state of New Mexico [24]. Annual variety trials with sorghum are conducted at ASC sites each year that evaluate cultivar yield to inform producers and seed companies about local variety selection. Biomass production data from the variety trials were used for DayCent model parameter calibration and validation for irrigated and dryland sorghum.

Experiments were initiated in Clovis, New Mexico (34.601 N, -103.214 W), where sorghum is currently grown under both dryland and irrigated conditions. Soil inputs to DayCent were acquired via NRCS Web Soil Survey using the Area of Interest (AOI) function in Web Soil Survey (WSS) which was limited by drawing a 3 km^2^ polygon around the Clovis ASC to include research sites [38]. Soil within this polygon was Olton clay loam (Fine, mixed, superactive, thermic Aridic Paleustolls), a slightly alkaline, well-drained soil with 0–1% slopes. Physical and chemical data for the input to DayCent were acquired for 0–180 cm depth (S1 Table).

### Current and future climatic conditions

DayCent requires daily weather inputs of minimum and maximum air temperature and precipitation. Historic weather data for Clovis, NM was obtained via Daymet V3.0 [39] from 1980–2015. These data were used in the calibration-validation steps, spin-up period and current climate simulations. Future weather files were taken from Phase 3 of the Coupled Model Intercomparison Project [40]. These projections were then statistically downscaled [41]. This was done with an asynchronous regional regression downscaling method using piecewise linear regression to bias correct and downscale global model results to a specific locale. The quantiles for each segment of the piecewise regression are derived from breakpoints in the rank-ordered data distribution rather than selected *a priori* (such as quartiles or deciles). This method is advantageous in the context of understanding ecosystem response to future weather because residuals in the tails of each distribution are minimized, and extremes are better represented.

All future weather data were derived using IPCC emissions scenario A1B which assumes technological improvements and a future with balanced fossil and non-fossil energy [42]. Five future weather models were selected based on the completeness of daily weather data, predictive power related to net primary productivity, carbon cycling, and similar spatial resolution (Table 1). While these weather projections were chosen for similarity on those criteria, there was still considerable variability among models for daily temperature and precipitation; hence the need to evaluate results from a suite of possible future scenarios related to sorghum yield and biogeochemical responses.

**Table 1:** Sources of daily future weather inputs to DayCent model.

Model	Supporting Institution	Link to Source
CGCM3.1	Environment Canada (Canada)	http://www.cccma.ec.gc.ca/data/cgcm3/cgcm3.shtml
CNRM-CM5	Centre National de Recherches Meteorologique (France)	http://www.cnrm.meteo.fr/spip.php?article126&lang=fr
ECHO-G	Meteorological Institute of the University of Bonn (Germany); Institute of KMA (Korea)	http://coast.gkss.de/staff/wagner/midhol/model/model_des.html
Hadgem	Met Office (United Kingdom)	http://www.metoffice.gov.uk/research/modelling-systems/unified-model/climate-models/hadgem3
PCM	Los Alamos National Laboratory (USA)	http://www.cgd.ucar.edu/pcm/

Global circulation models used to generate daily weather data under a future climate scenario (IPCC Scenario A1B). These models were chosen based on their completeness of daily weather data, predictive power of net primary productivity and carbon cycling, and similar spatial resolution. Links provided are to source information explaining the GCM’s in greater detail from the progenitors of the models.

### DayCent calibration and validation

DayCent is a daily time-step, process based biogeochemical model [37]. Parameters for sorghum had been previously developed for DayCent and the model has performed well in simulating field-measured soil temperature, water content and soil organic carbon (SOC) with sorghum in Texas [43]. Default sorghum growth parameters were compared to reported yield to determine baseline model performance. Model runs were made by scheduling management (date of planting, cultivation, irrigation, fertilization and harvest) in accordance with the published field trials. Variety yield trials were conducted by NMSU ASC by planting sorghum on fields that had been under fallow in the previous year. To simulate this in the model, two sets of runs were made in alternating years in which plots were either under fallow or in sorghum production to give a modeled data set for sorghum every year from 2010–2015, i.e., a simulation with 2010 sorghum, 2011 fallow, 2012 sorghum; a second simulation with 2010 fallow, 2011 sorghum, and so on. Model output was compared to field data after field data were converted from g C ∙ kg^-1^ to Mg C ∙ ha^-1^, assuming aboveground C concentration of sorghum to be 43.47% [44].

Model performance compared to field data was evaluated with the Nash-Sutcliffe statistic *E*:

$$E=1-\frac{{\sum \left(measured-modeled\right)}^{2}}{{\sum \left(measured-measurement\;mean\right)}^{2}}$$

This statistic ranges from negative infinity to 1.0. A value of 0.0 represents model fit to observed data equals to the mean of all observed data, and a value of 1.0 represents exact correspondence between modeled and observed data [45]. Thus, negative values represent poor model fit, and positive values suggest the model predicts measured values better than the mean of observed values [45].

**Fig 2:**
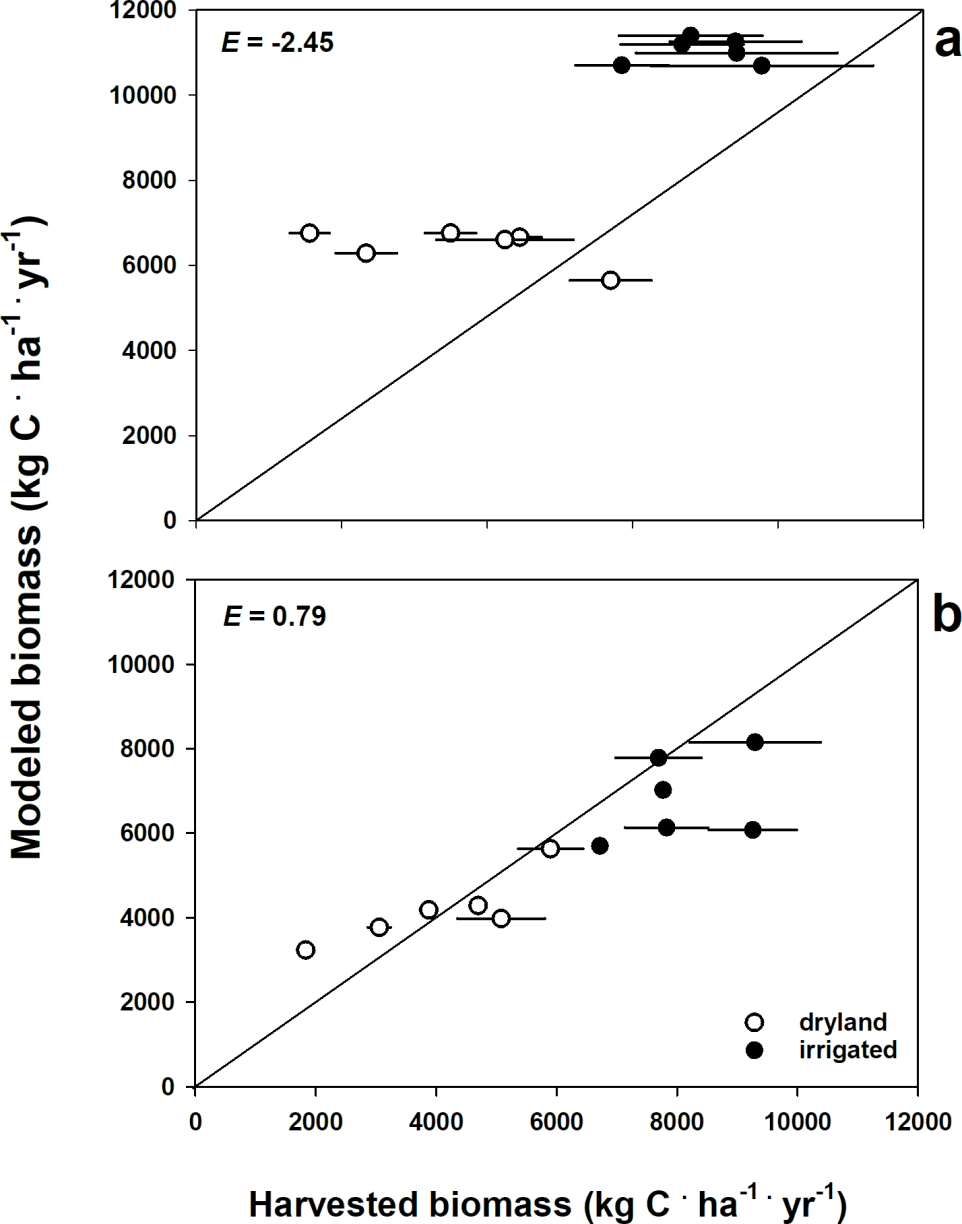
DayCent calibration for sorghum. Modeled aboveground biomass C at harvest compared to reported forage yield at the New Mexico Agricultural Science Center at Clovis from a) default crop growth parameters for sorghum and b) predicted sorghum yield following statistical calibration of crop growth with the parameter optimization software PEST.

An initial desire to include all available field data was confounded by the wide range of yield values reported for the various cultivars harvested each year, and default model settings consistently over-predicted yield (Fig 2; *E* = -2.45 for default versus all field data). Manual calibration of two parameters with strong controls on yield at harvest (prdx [1] and wscoeff [1,1]; crop response to light and a water stress multiplier, S2 Table) resulted in better model agreement with field trial data for the five highest yielding cultivars reported each year (*E* = 0.76). Thus, manual calibration proved sufficient for selecting model inputs for a high-yield cultivar.

However, we wished to improve DayCent performance for the range of observed sorghum cultivar biomass production. Further calibration was performed via an inverse modeling approach, by running DayCent within the parameter estimation software PEST [46]. This approach has been successfully employed for improving DayCent’s predictive power of C and N cycling in corn systems, where PEST lowered the sum of weighted squared residuals of the model by 56% compared to default settings [47]. Furthermore, the study [47] highlights the parameters most sensitive to change with respect to yield. As both sorghum and corn are annual crops it is a reasonable assumption that parameters with the greatest control on biomass production will be the same for both crops in DayCentand knowledge of sensitive model parameters prior to computing optimal parameters facilitates efficient calibration that is performed in a biologically meaningful way [48].

PEST estimates optimal parameters with a non-linear regression method based on least-squares minimization [47, 49]. The model to be calibrated is run within PEST so that the PEST code systematically changes the model inputs, runs the model (DayCent), reads model output from each run, and calculates model fit as the weighted least squared difference between observation data and model data [46]. A detailed description of PEST calibration of DayCent is found in [47].

Observation data for PEST calibration was the ASC sorghum yield. Dryland and irrigated yield data for 2010, 2012, and 2014 were used as observation comparisons for PEST. We set the calibration simulations to run the model 20 times per optimization iteration. The largest change from initial to optimized parameter values were observed for controls on growth due to response to solar radiation inputs, temperature response curves and water stress on potential growth (S2 Table). The resulting parameter adjustments were compared to the entire ASC data set (2010–2015) where that calibration improved DayCent fit to the observed data across the entire range of low and high yield sorghum varieties (*E* = 0.79), and for sorghum grown under both dryland and irrigated conditions (Fig 2).

### Simulation experiment

An initial 1970 year “spin up” period (year 0 to year 1970) was run in the model to establish soil C stocks and historic conditions for the site prior to our years of interest in the simulation experiments. This portion of New Mexico is on the southwestern edge of the Great Plains and is historically dominated by shortgrass prairie vegetation. For the spinup period, vegetation was modeled as shortgrass steppe. All simulations described hereafter began in 1971 as extensions of the spin-up period, thus all experimental runs began with identical soil conditions.

Nitrogen application rates (84 and 173 kg N ∙ ha^-1 .^ yr^-1^) were selected based on fertilizer quantity used in ASC field trials. Irrigated sorghum received more than twice the fertilizer compared to dryland sorghum in the field trials, making it impossible to test for main effects of N or irrigation alone from the field data. Previous work on corn suggests that when N application is split into multiple doses, NUE increases and reactive N losses are minimized [32, 50]. We therefore simulated different N application timing to sorghum, by adding all N at the time of planting (“basal” application), or applying 33% at planting and the remaining 67% at 30 days after planting (hereafter “split”).

Long periods of drought expected under future climatic scenarios might necessitate irrigation, but then pulses of unpredictable, intense rain, if following irrigation, could compromise production. Irrigation as a management tool will also be complicated by increases in precipitation variability which is an expectation of climate change in the southwestern US, during sensitive parts of the sorghum life cycle, such as nearing exponential growth, or following fertilizer application. Irrigation was applied in DayCent by setting water input quantities in ‘irri.100’ files. Irrigation amount was specified based on reported values for monthly water additions at the Clovis, NM ASC site during the years used for model validation. The irrigation routine added water weekly in the specified amounts.

The last component of the simulation experiment was to perform model runs using current (1971–2015) or future (2016–2065) weather data. Simulations were extended from the spin-up (0–1970) through current time periods, and weather files were changed in the batch code to future models beginning in 2016. As an example, a model run using the CGCM3.1 weather input would begin as an extension of the spin-up (years 0–1970), run with historic weather inputs for Clovis, NM from 1971–2015 and then the model would switch to using future weather downscaled from CGCM3.1 for years 2016 to 2065. Because a suite of models was used for future weather, the example above was repeated 5 times, once for each separate downscaled future weather file (Table 1), but all future runs were extended from the same management specific (N addition and irrigation) current weather runs which were extensions of the spin-up periods. Output data were split into current and future climatic conditions periods, with the years 1995–2015 that used historic weather designated as “current” and output from 2045–2065 designated as “future”. Future climatic conditions were parsed in this way to bracket the year 2050, which is often used as a benchmark because future weather uncertainty increases with temporal distance from the present [42].

### Statistical analysis

Input effects on sorghum productivity and biogeochemistry were evaluated by examining absolute changes in model output as well as scaling by inputs in this system. The overall experiment was a factorial design, with simulations run for every combination of irrigation (dryland versus irrigated), N application rate (low versus high), N application timing (basal versus split) and under current and projected future climate. We focus on output variables from DayCent related to sorghum production (above- and belowground biomass C), greenhouse gas flux (N_2_O emissions, CH_4_ oxidation, CO_2_ production from heterotrophic respiration), N leaching and changes to soil C pools.

Three-way ANOVA’s were run to test for main and interactive effects of N application rate, N timing and irrigation. Separate models were run for results from current climate and for each set of results from individual future weather simulations. Current and future weather results were compared with 1-way ANOVA to test for the main effect of climate. Correlation analysis was performed to relate aspects of daily weather to biomass accumulation. Analyses were performed using the ‘Aov’ and ‘cor.test’ functions in R [51, 52]. Fligner-Killeen test was used to test for homogeneity of variance. Tukey’s post-hoc test was run following ANOVA’s and statistical significance was assumed at α = 5%. When reported, effect size is the relative percent difference between treatments or groups.

In addition to examining main effects of N application rate, N timing and irrigation in the model, we also scaled annual production and emissions by input to determine the gain in biomass or change in emissions due to per unit increases in N addition and irrigation. Grain was proportional to aboveground biomass C at harvest, but not included in these analyses. Biomass units in the following equation are g C ∙ m^-2^. Model runs with zero additional N fertilizer, and under dryland conditions were run for current weather and the 5-model suite of future weather. Nitrogen effects on biomass (BN) or N_2_O emissions (EN) were calculated by subtracting the zero N biomass from the biomass of N addition runs, then dividing the result by the N application rate (8.4 or 17.3 g N ∙ m^-2^):

$$BN=\frac{Aboveground\;biomass\;at\;N\;rate-Aboveground\;biomass\;N\;zero}{N\;rate\;(g\;N\;∙m^{-2})}$$ Eq 1

$$EN=\frac{N2O\;emissions\;at\;N\;rate-N2O\;emissions\;at\;N\;zero}{N\;rate\;(g\;N∙m^{-2})}$$ Eq 2

Then, additional effects on biomass or emissions due to irrigation were accounted for by taking the difference between identical N additions (equal rate and same timing) that were simulated under irrigated and dryland conditions and dividing that result by the quantity of water added via irrigation (irrigation mm H_2_O). Although irrigation additions are scheduled on a per-week basis in input files to DayCent, these water additions appear in DayCent output files as additional daily precipitation. Thus, daily precipitation must be accounted for to quantify irrigation inputs. The input scaled biomass (ISB) and input scaled emissions (ISE) are calculated as follows:

$$ISB=\frac{BN\;irrigated-BN\;dryland}{total\;irrigation\;H_{2}O\;(mm)}$$ Eq 3

$$ISE=\frac{NE\;irrigated-NE\;dryland}{total\;irrigation{\;H}_{2}O\;(mm)}$$ Eq 4

The resulting value is the yield (or emission mass) expressed as a function of exogenous input intensity, i.e., change in biomass or gas emissions per unit of fertilizer above background N levels in soil and and irrigation (g C ∙ g N^-1 .^ mm H_2_O^-1^ or g N_2_O-N ∙ g N^-1 .^ mm H_2_O, respectively).

## Results

### Irrigation effects

**Fig 3:**
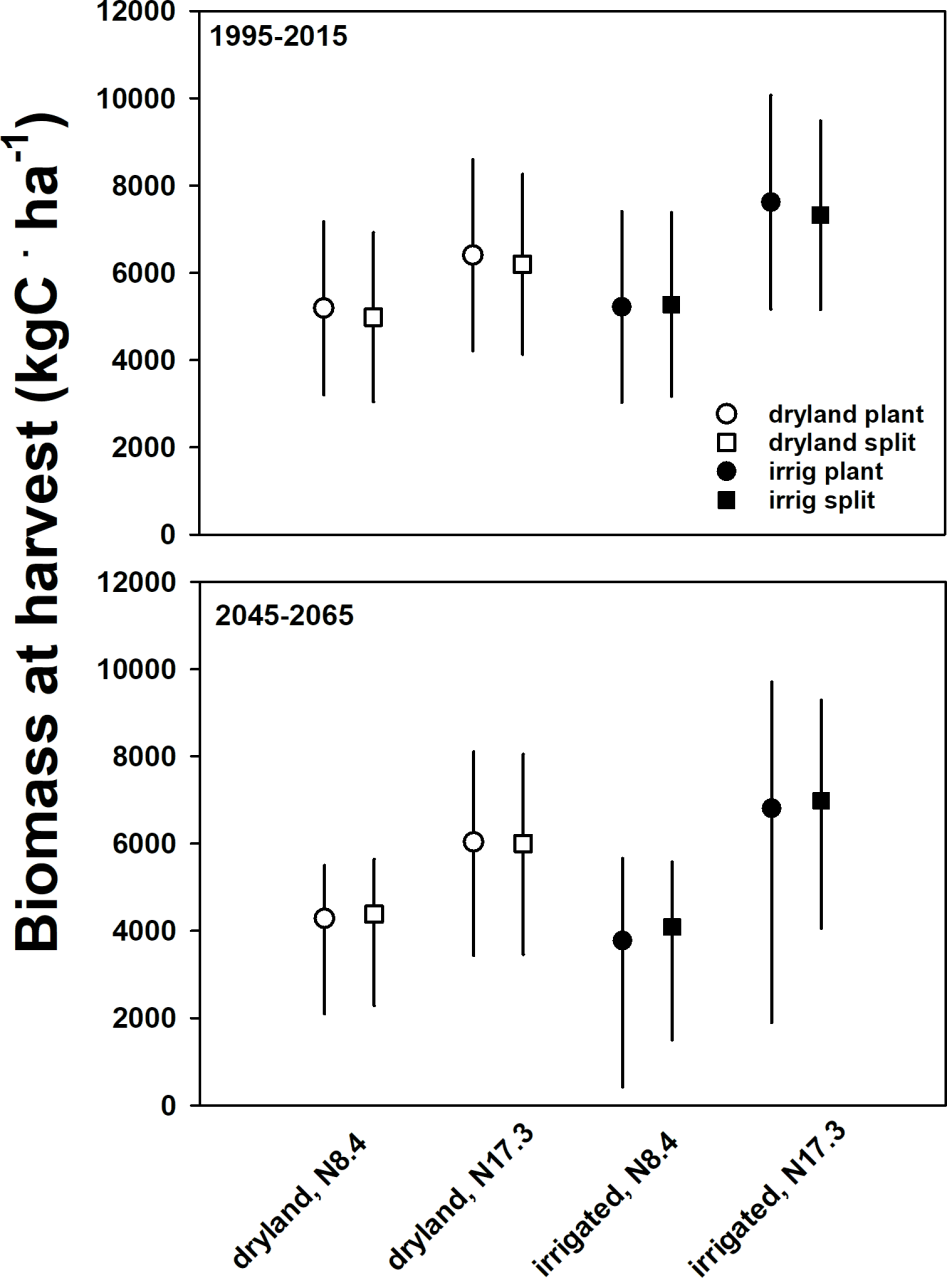
Sorghum biomass response to treatments in DayCent. Simulated sorghum biomass C in response to two N application rates, variable N timing and irrigation vs. dryland under current (1995–2015) or a suite of future (5 models; 2045–2065) weather. Error bars for current weather represent ± 1 SD, or the range of values from simulation values across a suite of future weather inputs.

Contrary to our expectations, irrigation did not significantly increase aboveground biomass (Fig 3). There was a 19% increase in aboveground biomass of irrigation at high N application rate, but only a 3.2% increase at low N under current weather. The two model-years with the lowest precipitation (212 and 264 mm, respectively) showed the largest increase in biomass under irrigation (90–150% yield increase). The mean irrigation effect across all fertilizer treatments was 12% for current climatic condition (effect size range -1% to +20%) and 5% for future climatic conditions (effect size range -80% to +20%). The only N input combination with positive irrigation effect (effect size range = +15 to 17%) on biomass under future weather was split N at high N rate (Fig 3). While not formally significant (*P* = 0.06), root production was positively related to irrigation under current climate (8% greater than dryland). There was a wide range of irrigation effect on root mass when considered separately from N rate and N timing under future weather (-71% to 94%), a consequence of DayCent increasing allocation to belowground plant C pools under water or N stress.

**Fig 4:**
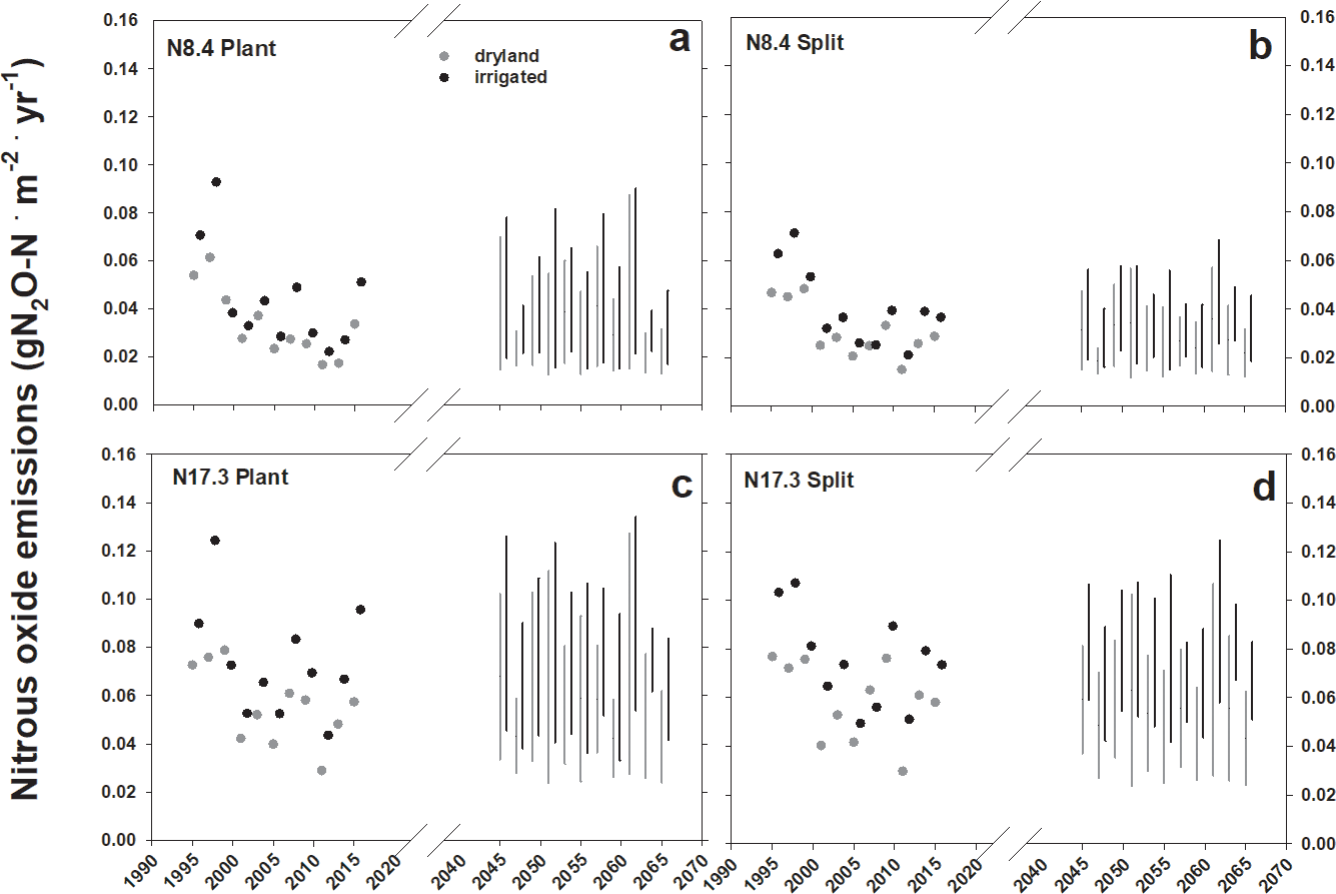
Simulated N_2_O emissions under current and future climate. Simulated nitrous oxide emissions from sorghum cultivated with four nitrogen management scenarios under irrigated (black circles and bars) and dryland (grey circles and bars) agriculture, for current (1995–2015) and future (2045–2065) weather. Symbols are cumulative N_2_O emissions per year for current weather, and bars represent the range of emissions from five climate models. Nitrogen management scenarios are as follows: Nitrogen application rates of 8.4 g N ∙ m^-2^ during A) single application of nitrogen fertilizer at time of planting (basal) or B) split nitrogen applications at 33% time of planting and the remaining 67% at 30 days post planting (split). Additional results are for fertilizer application rates of 17.3 g N ∙ m^-2^ at C) planting or D) split application.

Irrigation increased per area N_2_O emissions under historic weather (Fig 4; F_1,88_ = 7.30, *P* < 0.01). Irrigation caused a 1–11% stimulation of heterotrophic respiration (R_H_) at low N rate relative to dryland, but a 14–20% increase in R_H_ at high N rate under current climate. The N_2_O emissions and R_H_ were significantly higher under irrigation in four of the five future simulations (S3 Table). The N_2_O emissions under future climatic conditions trended toward larger variation at the high N ratesand higher emissions under irrigation (Fig 4). Methane oxidation rates were significantly depressed by irrigation in the simulations under current climate conditions (F_1,88_ = 35.41, *P* < 0.001). All future climate models showed the same pattern for lower CH_4_ oxidation rates under irrigation (S3 Table).

### N fertilization rate effects

**Fig 5:**
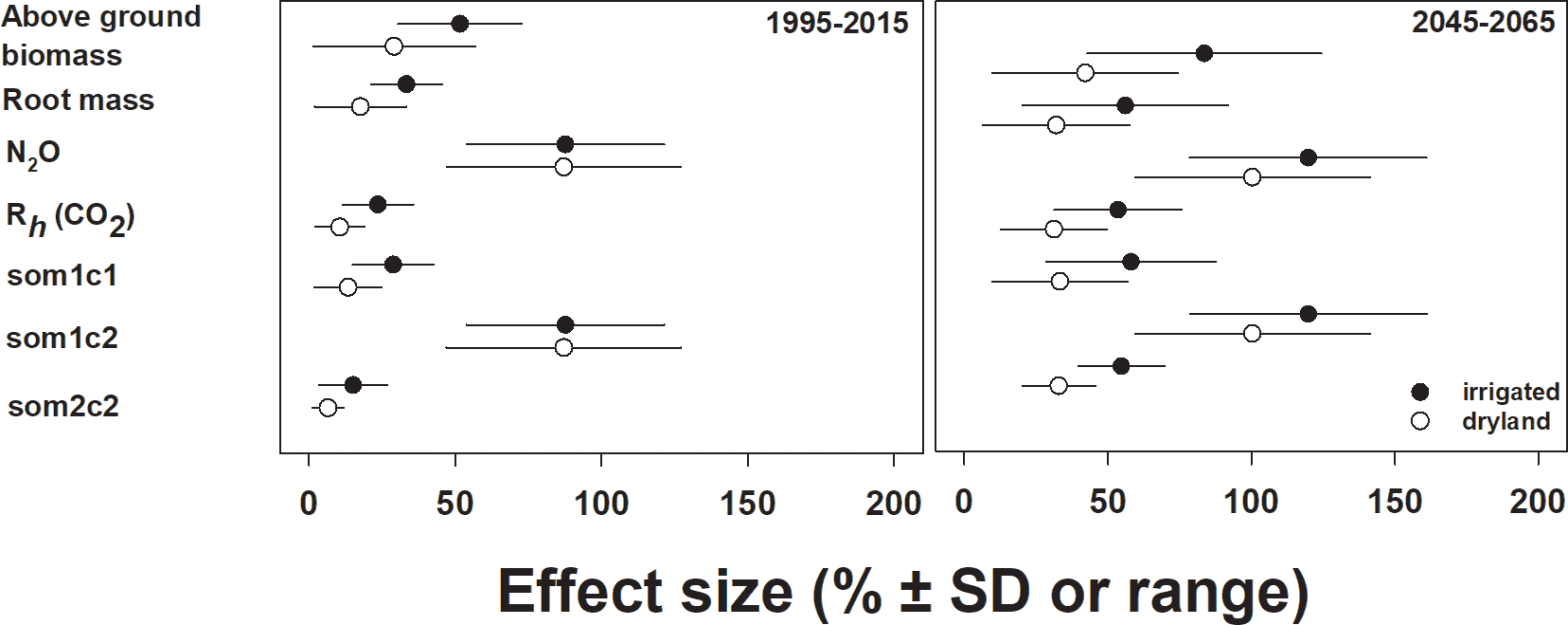
Effect of N inputs to sorghum-system biogeochemistry. Proportional effect (%) of increasing N inputs by 105% from 8.4 to 17.3 g N ∙ m^-2^, on biogeochemical variables from DayCent simulations for irrigated or dryland sorghum. Error bars for current weather represent ± 1 SD, or the range of values from simulation values across a suite of future weather inputs. Variables for soil organic carbon pools: ‘som1c1’ = surface active SOC, ‘som1c2’ = soil active C pool, ‘som2c2’ = soil slow C pool.

Changes in sorghum biomass, trace gas production and soil organic C due to management all showed a stronger response to N rate than to irrigation (Fig 5). Sorghum biomass under current climate was significantly higher at increased N fertilization rate (Fig 3 and S2 Table; F_1,88_ = 15.43, *P* < 0.001). When main effects of irrigation and N rate were measured alone, increasing N rate from 84 to 173 kg N ∙ ha^-1^ increased aboveground biomass by 52% under irrigation and 23% for dryland condition (Fig 5). We did not observe any other main effects or interactions affecting biomass under current climate. Contrary to our hypothesis, there was no effect of N application timing on biomass under current climatic conditions (*P* > 0.50).

Fine root production was significantly correlated with C allocation to the slow (10–50 year turnover) soil organic C pool (r = 0.63, n = 88, *P* < 0.001). This relationship followed the gradient of management intensity, with the highest belowground C inputs coming from simulations under high fertilizer application and irrigation which follows the pattern of higher root production under those treatments. This trend held for future climate simulations, even though gross fine root production and soil C allocation were significantly lower than under current weather. Juvenile roots growth was positively stimulated by increased N in the model (F_1,88_ = 10.31, *P* < 0.001). We observed a greater effect of irrigated vs. dryland on fine root production at low N rate with split N application (+13% split versus 1% effect with basal N). Split N applications at high N rate increased root growth by 19% under irrigated condition compared to 17% for basal application.

Soil slow C pool changes in the simulations were calculated as the difference between last and first model-years considered “current” or “future” climate. Current climate C pools substantially declined for all N rate, N application method and irrigation treatments (Table 2). Future C pools tended toward modest increases, with the largest mean gains across weather models for sorghum simulated under irrigated conditions with low basal N rate (Table 2). High N fertilization under current weather increased gross nitrification (F_1,88_ = 113.2, *P* < 0.001) as well as per area N_2_O emissions (F_1,88_ = 40.4, *P* < 0.001). Increasing N rate increased R_H_ in all future weather simulations (S3 Table).

**Table 2:** Changes in 10–50 year soil organic carbon turnover in response to modeled management in sorghum.

			*Current Climate*		*Future Climate*	
Irrigation	N applied (g N ∙ m^-2^)	N timing	SOC Change (%)	Max C Loss	Max C Gain	Average Change Across Models
Dryland	8.4	BASAL	-544.90 (-66%)	-24.37 (-11%)	31.27 (16%)	9.36 (6%)
		SPLIT	-446.29 (-63)	-25.81 (-12)	25.00 (14)	2.49 (2)
	17.3	BASAL	-505.60 (-61)	-108.24 (-37)	31.49 (13)	-16.82 (-5)
		SPLIT	-398.02 (-56)	-35.26 (-13)	31.40 (14)	-10.89 (-4)
Irrigated	8.4	BASAL	-437.69 (-63)	-21.52 (-11)	72.78 (54)	18.60 (13)
		SPLIT	-441.19 (-64)	-14.40 (-7)	50.83 (33)	12.83 (8)
	17.3	BASAL	-354.09 (-50)	-23.75 (-8)	71.33 (31)	13.72 (6)
		SPLIT	-363.95 (-52)	-22.07 (-7)	37.30 (15)	7.30 (3)

Soil organic carbon slow pool (10–50 year turnover) changes in DayCent simulations from the first model-year to the last for time periods considered (current = 1995–2015, future = 2045–2065). N application method refers to all nitrogen applied at planting (BASAL) or 67% applied at planting and 33% applied at 30 days post-planting (SPLIT). Minimum and maximum values for future simulations are from the pool of 5 downscaled future weather simulations, and average change is the mean. Absolute values are g C ∙ m^-2^, percent change in parentheses.

### Interactive effects

There was a significant N rate by irrigation interaction for both R_H_ and N_2_O emissions for four of the five future weather runs (S3 Table). Respiration losses were lowest for dryland simulations under low N rate, and 60% greater losses resulted from irrigation at high N rate. Soil slow organic C pool followed a similar trend, with all future simulations showing a significant N rate x irrigation interaction, and the largest C pools were at high N rate under irrigation. The lowest slow organic C pools were simulated under irrigation and low N rate (S3 Table).

We examined possible legacy effects of sorghum production on N_2_O flux by comparing high input (irrigation and high N rate) compared to low input (dryland and low N rate) years following sorghum. Under current climatic conditions, there was a 14% decline in N_2_O emissions from high input relative to low inputs, but under future weather, N_2_O emissions was 49% higher from increased inputs to sorghum.

### Climate effects

As stated in the methods, independent weather models used as projections of future weather must be considered independently for statistical analysis. Model independence was evident as three of the five future weather models predict statistically significant increases in total annual precipitation for Clovis, NM, but the other two models project no change in total precipitation (Fig 1). There was consistency across the models where harvested biomass was lower under future than to current weather (Fig 3).

**Fig 6:**
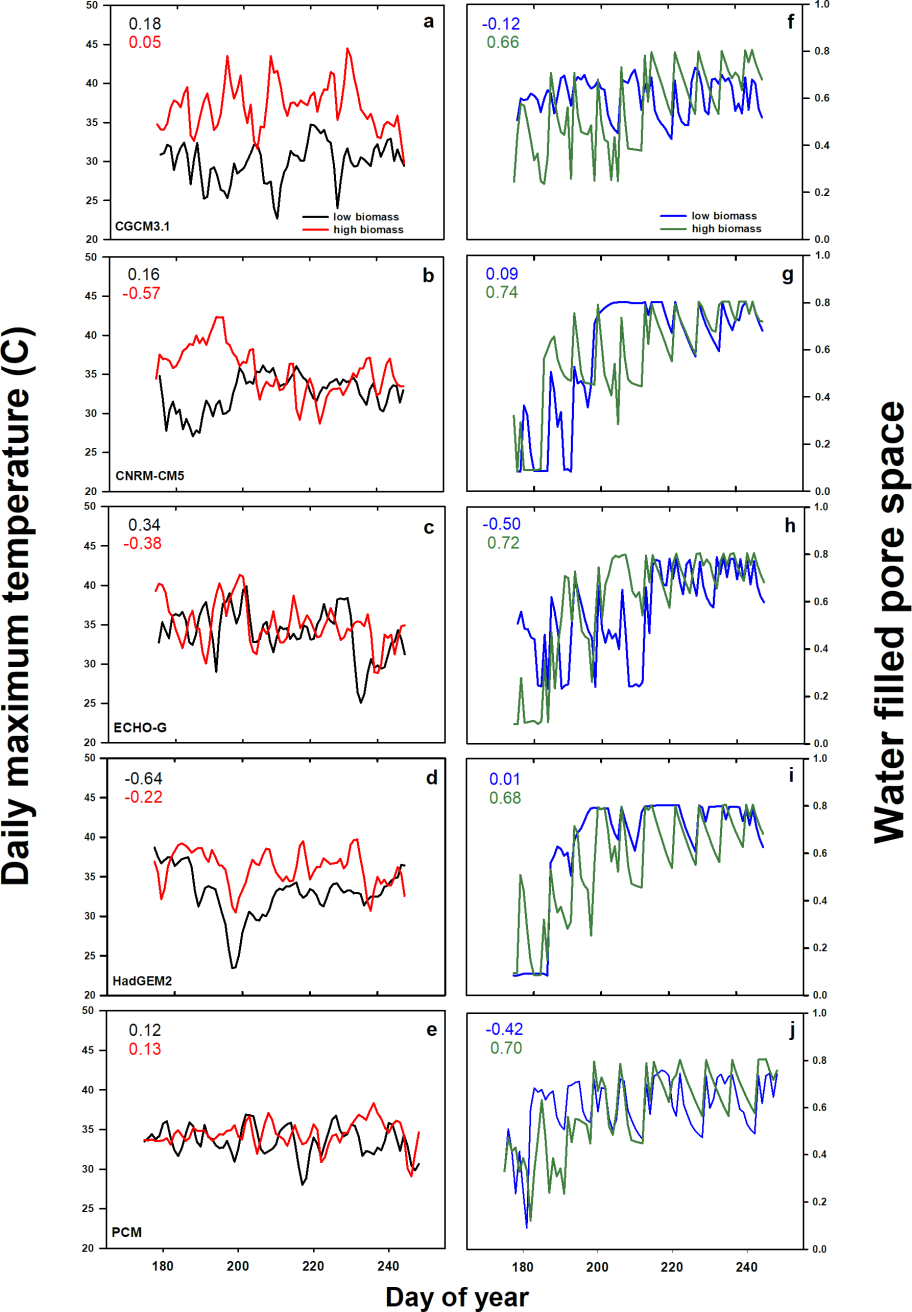
Future weather and soil water explain sorghum yield extremes. Future growing season weather and soil water as explanations of extremes in sorghum yield. Lines are from extreme low and high biomass years in simulations. Daily maximum air temperatures (Tmax) (a-e) and variations in growing season water filled pore space (wfps) (f-j). Global circulation model from which downscaled weather files were generated in the lower right of Fig a-e, and correspond to paired wfps Fig f-j. Colored values in the upper left of each panel represent correlation coefficient for correlations of Tmax or wfps with daily biomass increment for high and low biomass simulations.

Simulation years with extreme values for harvested biomass were examined more closely to determine model mechanisms driving lower yield under future climatic conditions. All instances of the highest and lowest sorghum yield-year relationship for each weather model occurred under irrigation. Fig 6 displays growing season daily air temperature and soil water content for the highest and lowest productivity years for each weather model. Results from the highest yielding simulation years showed negative correlations between sorghum production and daily maximum air temperature (Tmax) (Fig 6), and positive correlations (*r* = 0.66–0.74) between production and water filled pore space (wfps) (Fig 6).

High growing season temperatures did not necessarily compromise yield in the model. Negative correlations between air temperature and production in high yielding years were observed when there were many warm days at the beginning of the growing season, and when the growth increment of the plant per day was still relatively low (Fig 6B–6D). The relationship between sorghum production and wfps in DayCent was positive when soil water declined in between irrigation events, but this was dependent on when dry-down occured during the growing season (Fig 6). Production was compromised when soil water declined steeply to <0.30 wfps, as the plant was entering a rapid growth phase near day 210 (Fig 6H). Conversely, high wfps of >0.70 also reduced production, as the lowest yielding simulation years for three future weather models occurred when wfps remained high for 10 days or longer (Fig 6F, 6G and 6I). High production co-occurs with high wfps, if that soil condition occurs later in the growing season (Fig 6F).

### Input-scaled biomass and N_2_O emissions

**Fig 7:**
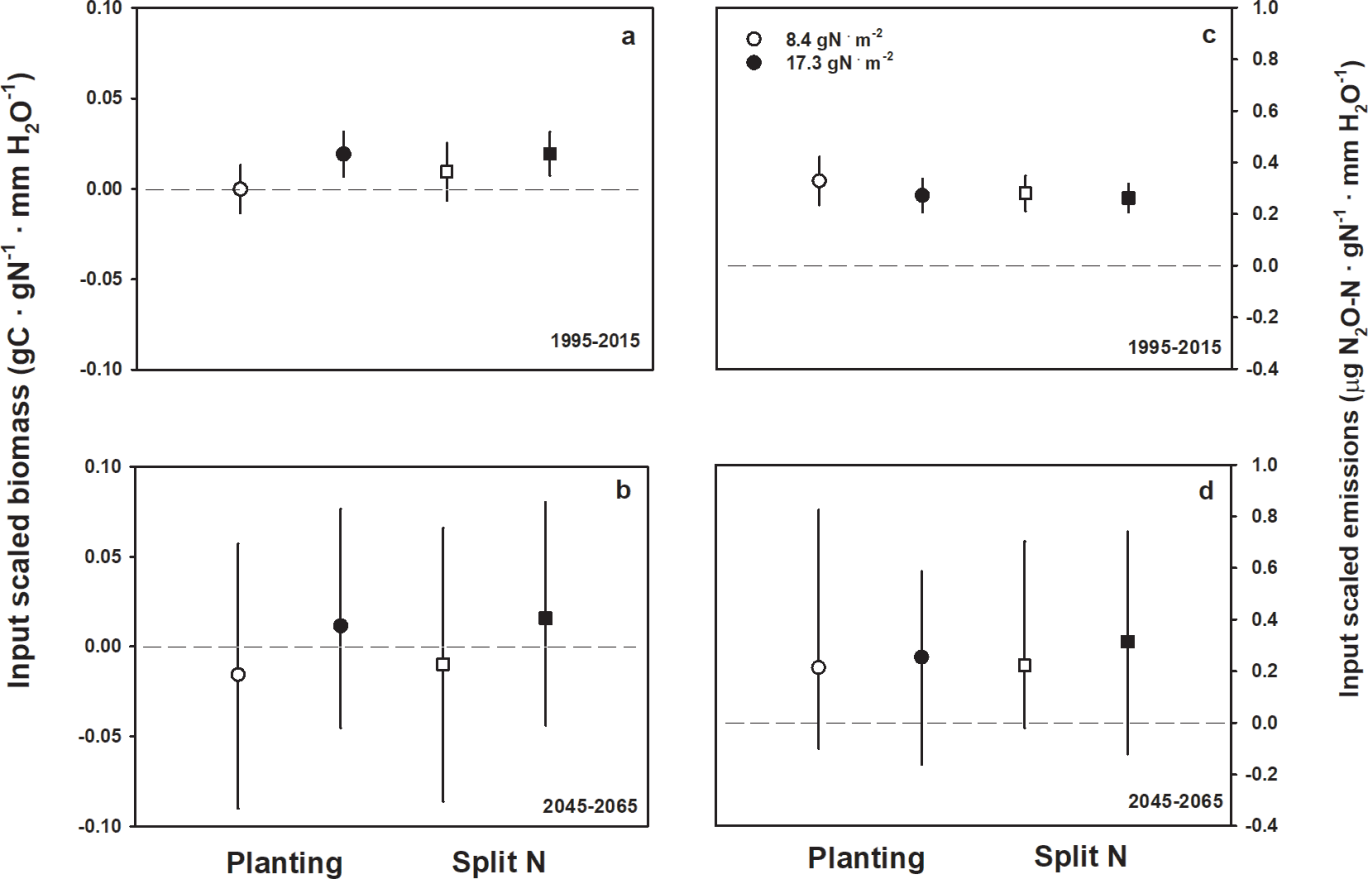
Input-scaled sorghum biomass C and N_2_O emissions. Simulation results for input scaled biomass C (panels a and b) and nitrous oxide emissions (c and d) for sorghum grown under current and future climate. Open symbols are for simulations at 8.4 g N ∙ m^-2^, closed symbols represent 17.3 g N ∙ m^-2^ fertilizer rates. Scaling is accomplished by considering fertilizer mass (g N ∙ m^-2^) and irrigation (mm H_2_O) used to produce a given unit of aboveground biomass or nitrous oxide. Scaling equation detailed in methods, points above or below dashed zero line represent increase or decrease in biomass or emissions due to fertilization or irrigation. Points are mean value and error bars are SEM for current weather simulations (a and c) or the range of simulation values across a suite of future weather inputs (b and d).

Scaling biomass by N rate and water inputs showed a slight decline in ISB under current climate when irrigated sorghum was grown under basal, low N rate (Fig 7A). In contrast, irrigated sorghum under split N application showed an ISB of ~10 mg C ∙ g^-1^ N ∙ mm^-1^ H_2_O at low N, and 20 mg C ∙ g^-1^ N ∙ mm^-1^ H_2_O at the high N rate (Fig 7A). Under future climatic conditions, mean ISB for high N rate was positive and negative for low N inputs, but substantial variation existed among the future weather models (Fig 7B).

We calculated biomass-scaled emissions to compare with our input-scaling framework. Simulations resulted in significantly higher biomass-scaled emissions with high N rate in the current weather simulations (F_1,88_ = 8.35, *P* < 0.01). There was no statistical difference in biomass-scaled N_2_O between high and low N treatments in the future simulations for three of the five weather inputs, even though N rate had a significant, positive effect on both aboveground biomass and N_2_O emissions for all future simulations (S3 Table). Our novel framework of ISE were lower for sorghum simulations using current weather with the higher N rate and irrigation (Fig 7C). We calculated a wide range of ISE values for future climatic simulations, but there was a general trend for greater ISE with high N rate, and greater ISE with split N rate (Fig 7D).

**Fig 8:**
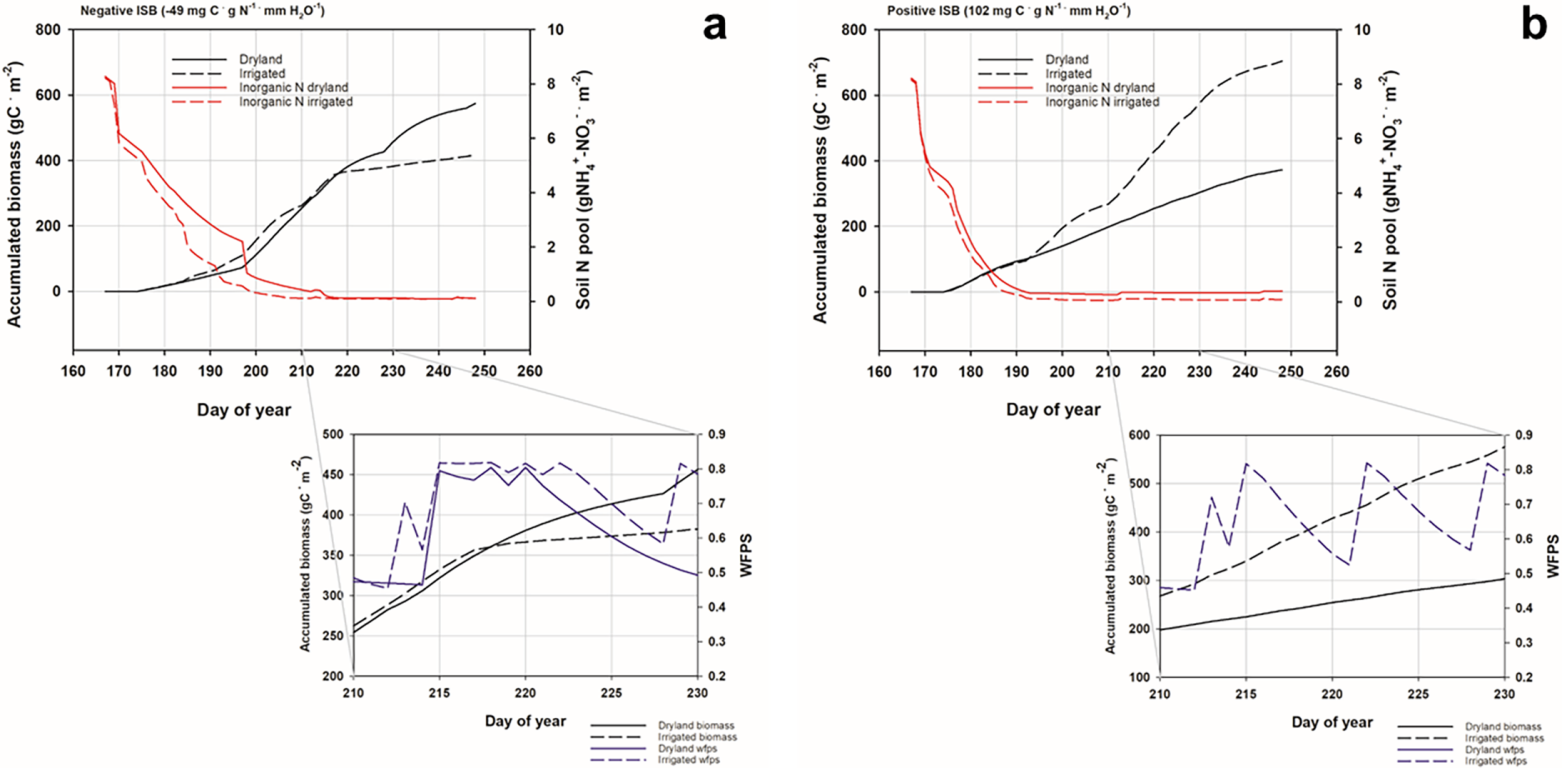
Modeled sorghum growth and soil N pools. Daily modeled sorghum growth trajectories in relation to inorganic nitrogen pools in the simulation year with the most a) negative input scaled biomass (ISB) and b) largest positive ISB. Fig expansions to show daily water filled pore space overlain on biomass at the point in growing season where plants enter exponential growth in the model.

To find a mechanistic explanation for how high inputs resulted in lower yield, we examined biomass over a growing season compared to daily inorganic N and soil water contents from dryland and irrigated sorghum in the model years with the largest and smallest (most negative) ISB. Growing season temperature for the highest ISB was erratic, with maximum temperatures varying more than 10°C between days, from day 210–220 and did not offer a satisfactory explanation of model behavior. Examining changes in soil inorganic N pools for the year with negative ISB shows that under dryland condition a greater reserve N pool is present prior to a shift in the growth trajectories. The Fig expansion shows that an increase in water-filled pore space from precipitation in the dryland treatment coincides with a sharp decline in soil inorganic N spurred by increased wfps and plant uptake at day 217 (Fig 8A). Conversely, the year with highest ISB was characterized by warm-dry conditions which lowered water filled pore space following irrigation, drawdown of soil N, and coincident with a sharp increase in the slope of biomass through day 230. This led to the accumulated biomass stimulation (and high ISB) observed under irrigation in that model year (Fig 8B).

## Discussion

The driving factors in these simulation results were related to climate change and agriculture: N management and precipitation variability. Nitrogen had the largest effect on annual and decadal patterns in sorghum production, greenhouse gas emissions and changes in soil organic C, but irrigation and specifically soil water, were responsible for fine-scale within season results. Irrigation had the largest effect on above- and belowground biomass C only at high N rate or in model years with very low precipitation. Irrigation did increase N_2_O and CO_2_ emissions, but gas flux was more strongly related to N fertilizer under both current and future climatic conditions (Fig 5).

One of the most commonly cited weather predictions under future climate is changes to precipitation intensity and frequency [42]. This variability was accounted for in our experiments by utilizing a suite of future weather projections and was important in driving biomass and N_2_O emissions patterns during the growing season. Soil N pools, irrigation and precipitation worked in concert to increase the slope of growing season biomass C accumulation on the soil modeled here. Sorghum responded in the model with vigorous growth when irrigation timing was staggered with precipitation. This is intuitive as optimal wfps for crops is near 0.5 and N losses via N_2_O emissions in DayCent increase exponentially between wfps 0.55–0.90 [53, 54].

It is unsurprising that maintaining optimal soil water and high temperatures while retaining soil N would spur sorghum growth, but how are those conditions consistently achieved and the converse avoided? It is unlikely that a producer would knowingly irrigate after a precipitation event, however, a farmer has no control over precipitation following irrigation, which can severely reduce production if soils become waterlogged and anoxic [55]. The ISB and ISE framework guided a deeper dive into the simulations and showed that even in an arid system, excess water leads to under production even at high N rate.

Ramirez-Villegas et al. [56] took a continental scale modeling approach to estimate sorghum under future climatic conditions. The authors suggested that future sorghum yields are most uncertain in areas predicted to be the most vulnerable to climate change where sorghum production is marginal [56]. The geographic scale of that work is much greater than our simulation study, but the EcoCrop model employed in that study does not incorporate soil physio-chemical parameters, or provide output for C and N fluxes. The DayCent simulations offer daily resolution for specific growing season weather-soil interactions that enhance or compromise sorghum production, namely prolonged high soil moisture, or water deficits between days 200–220 (Fig 6G & 6H). We were also able to make some predictions that while N_2_O flux variability rises with increased precipitation variability, splitting N in the simulations showed less variability in the future (Fig 4B & 4D). The N_2_O emissions are driven by the total quantity of N applied to a system, but understanding that gas flux from soil is a consequence of short-term interactions between N substrates to microbes, soil water and temperature and plant growth. Understanding the role of these interacting factors is necessary for a system approach to N_2_O mitigation.

As calculated here, ISB can only be presented for irrigated simulations because the metric relies on comparing production under different management practices. However, dryland biomass C simulations showed that some production is possible under those conditions. Indeed, explicit with a negative ISB is that dryland produced more biomass than irrigated land for a specific simulated year. It is worth considering that dryland systems could meet yield demands for certain crop components such as forage, especially given the added cost of irrigation if it is confounded by unpredictable rain. A suitable alternative would be precision irrigation when added water can be matched to specific plants experiencing water stress due to highly localized soil water conditions [57]. A conceptually similar idea with variable N application matched to individual plant need has shown promise in corn systems where reductions in overall fertilizer use have no impact on yield but significantly reduce both fertilizer use and N_2_O emissions [32, 50].

Correlations between fine root production and slower degrading pools of SOC suggest that years with high biomass production can increase SOC within a growing season. Over 20-year simulation periods, there were substantial losses of SOC under current weather in our simulations and some slight increases in SOC under future climatic conditions. Daily C accrual from sorghum roots is perhaps a function of available N and soil water status, such as we found for aboveground production. However, this is a knowledge gap with a clear need for increased field data to parse the fate of root C inputs from both biomass and exudates. Sorghum presents unique potential for exudate studies, as the allelopathic compound sorgoleone is produced from its roots, and only limited information exists for how soil microbiota respond to it [58]. Our inverse-modeling validation of production with field data should give confidence in these simulation results for biomass and N_2_O fluxes, but the potential for sorghum to induce significant influence on soil microbial communities via sorgoleone is high and warrants further investigation into the factors controlling the chemical’s synthesis and ecosystem level effects [59, 60].

## Conclusions

The DayCent model predicts that input intensity drives semiarid sorghum productivity and greenhouse gas emissions associated with its cultivation. Both sorghum production and N_2_O flux are strongly influenced by N rate and irrigation at annual and decadal time scales and less so by fertilizer timing. We found that DayCent predicts that shorter-term dynamics on the order of days within the growing season are strongly controlled by soil water content, and these predictions held under current and future climatic simulations. Scaling production and gas flux by exogenous inputs (N and water) shows that significantly greater biomass is not always predicted from high-intensity inputs and dryland sorghum production can be favored under specific conditions. These simulations help make the case that daily fluxes of soil water content and organic C inputs are critical to understand the annual and decadal controls on system-level production and N cycling, and to make management decisions that improve agro-ecosystem sustainability.

## Supporting information

S1 Table Soil physical and chemical parameters from Clovis, New Mexico used as input to the DayCent model. (DOCX)

S2 Table DayCent parameter value changes from default settings for sorghum to values calibrated with the parameter optimization software PEST. (DOCX)

S3 Table 3 way ANOVA results from DayCent simulations of semiarid sorghum production. (XLSX)
