# Supplementary material for: Water and nitrogen management effects on semiarid sorghum production and soil trace gas flux under future climate
Source: PLoS One. 2018 Apr 19;13(4):e0195782. doi: 10.1371/journal.pone.0195782 (PMC5908084; doi:10.1371/journal.pone.0195782)
Supplement: S1 Table — (DOCX) [file pone.0195782.s001.docx]

Table S1: Soil physical and chemical parameters from Clovis, New Mexico used as input to the DayCent model.

| Min Depth (cm) | Max Depth | Bulk Density | Field Capacity (Vol) | Wilting Point (Vol) | Percent Roots in Layer | Sand Fraction | Clay Fraction | Organic Matter (%) | Min VSWC | kSat  (mm ^.^ s^-1^) | pH |
| --- | --- | --- | --- | --- | --- | --- | --- | --- | --- | --- | --- |
| 0.00 | 2.00 | 1.54 | 0.35 | 0.24 | 0.01 | 0.34 | 0.31 | 0.03 | 0.20 | 0.90 | 7.10 |
| 2.00 | 5.00 | 1.54 | 0.35 | 0.24 | 0.04 | 0.34 | 0.31 | 0.03 | 0.20 | 0.90 | 7.10 |
| 5.00 | 10.00 | 1.54 | 0.35 | 0.24 | 0.25 | 0.34 | 0.31 | 0.03 | 0.20 | 0.90 | 7.10 |
| 10.00 | 20.00 | 1.54 | 0.35 | 0.24 | 0.30 | 0.34 | 0.31 | 0.03 | 0.20 | 0.90 | 7.10 |
| 20.00 | 30.00 | 1.46 | 0.36 | 0.25 | 0.10 | 0.34 | 0.39 | 0.01 | 0.05 | 0.27 | 7.70 |
| 30.00 | 45.00 | 1.46 | 0.36 | 0.25 | 0.05 | 0.35 | 0.39 | 0.01 | 0.05 | 0.27 | 7.70 |
| 45.00 | 60.00 | 1.46 | 0.36 | 0.25 | 0.04 | 0.35 | 0.39 | 0.01 | 0.05 | 0.27 | 7.70 |
| 60.00 | 75.00 | 1.45 | 0.35 | 0.24 | 0.03 | 0.35 | 0.39 | 0.01 | 0.05 | 0.27 | 7.80 |
| 75.00 | 90.00 | 1.45 | 0.35 | 0.24 | 0.02 | 0.35 | 0.39 | 0.01 | 0.05 | 0.27 | 7.80 |
| 90.00 | 105.00 | 1.42 | 0.35 | 0.23 | 0.01 | 0.36 | 0.38 | 0.01 | 0.00 | 0.27 | 7.90 |
| 105.00 | 120.00 | 1.42 | 0.35 | 0.23 | 0.00 | 0.36 | 0.38 | 0.01 | 0.00 | 0.27 | 8.10 |
| 120.00 | 150.00 | 1.49 | 0.33 | 0.23 | 0.00 | 0.38 | 0.39 | 0.00 | 0.00 | 0.88 | 8.20 |
| 150.00 | 180.00 | 1.50 | 0.33 | 0.23 | 0.00 | 0.38 | 0.39 | 0.00 | 0.00 | 0.88 | 8.20 |
