# Supplementary material for: Water and nitrogen management effects on semiarid sorghum production and soil trace gas flux under future climate
Source: PLoS One. 2018 Apr 19;13(4):e0195782. doi: 10.1371/journal.pone.0195782 (PMC5908084; doi:10.1371/journal.pone.0195782)
Supplement: S2 Table — (DOCX) [file pone.0195782.s002.docx]

Table S2: DayCent parameter value changes from default settings for sorghum to values calibrated with the parameter optimization software PEST.

| Parameter | Description | Units | Initial value | Lower bound | Upper bound | Calibrated values |
| --- | --- | --- | --- | --- | --- | --- |
| prdx(1) | Coefficient for calculating potential aboveground monthly production as a function of solar radiation outside the atmosphere | g C/m^2^/Langleys of shortwave radiation | 1.8 | 1 | 5 | 1.31404 |
|  |  |  | |  |  |  |
| ppdf(1) | Optimum temperature for production for parameterization of a Poisson Density Function curve to simulate temperature effect on growth | ˚C | 30 | 10 | 40 | 30.1854 |
|  |  |  |  |  |  |  |
| ppdf(2) | Maximum temperature for production for parameterization of a Poisson Density Function curve to simulate temperature effect on growth | ˚C | 35 | 30 | 45 | 35.184 |
|  |  |  |  |  |  |  |
| ppdf(3) | Left curve shape for parameterization of a Poisson density function cure to simulate temperature effect on growth | unitless | 1 | 0.5 | 10 | 1.5 |
|  |  |  |  |  |  |  |
| ppdf(4) | Right curve shape for parameterization of a Poisson density function cure to simulate temperature effect on growth | unitless | 1 | 0.5 | 10 | 0.5 |
|  |  |  |  |  |  |  |
| biomax | Biomass level above which the minimum and maximum C:element ratios of the new shoot increments equal pramn(1,1) | g biomass/m^2^ | 400 | 0 | 1000 | 400.008 |
|  |  |  |  |  |  |  |
| pramn(1,1) | Minimum C:N with zero biomass | unitless | 20 | 1 | 100 | 20.0895 |
|  |  |  |  |  |  |  |
| himax | Harvest index maximum (fraction of aboveground live C in grain) | unitless | 0.5 | 0 | 1 | 0.544894 |
|  |  |  |  |  |  |  |
| wscoeff(1,1) | Water Stress Coefficient used to calculate the water stress multiplier on potential growth based on the relative water content of the wettest soil layer in the rooting zone | unitless | 0.48 | 0.1 | 2 | 0.422868 |
|  |  |  |  |  |  |  |
| wscoeff(1,2) | See above | unitless | 8.5 | 0.5 | 20 | 8.39009 |
